# Platinum desensitization therapy and its impact on the prognosis of ovary high-grade serous adenocarcinoma: a real world-data

**DOI:** 10.3389/fimmu.2024.1346464

**Published:** 2024-01-19

**Authors:** Kemin Li, Rutie Yin

**Affiliations:** ^1^ The Department of Obstetrics and Gynecology, West China Second University Hospital of Sichuan University, Chengdu, Sichuan, China; ^2^ Key Laboratory of Birth Defects and Related Diseases of Women and Children (Sichuan University), Ministry of Education, Sichuan, China

**Keywords:** high-grade serous adenocarcinoma of the ovary, platinum allergy, platinum desensitization therapy, platinum-sensitive recurrence, prognosis

## Abstract

**Background:**

To examine the value of five-step platinum desensitization therapy in epithelial ovarian cancer

**Methods:**

A retrospective study was conducted on the high-grade serous adenocarcinoma of the ovary (HGSAO) patients who developed a platinum allergy during treatment and received desensitization therapy between January, 2016 and December, 2020. The logistic-regression was adopted to analyze the relationship between platinum desensitization therapy and prognosis in HGSAO patients.

**Results:**

92 HGSAO patients were included in the study. Among these, 35 patients (38.0%) experienced mild allergic reactions, 51 (55.4%) experienced moderate allergic reactions, and 6 (6.5%) experienced severe allergic reactions. The desensitization therapy was successful in 86 patients (93.5%). Six patients had desensitization failure, of which five experienced severe allergic reactions during desensitization. The logistic-regression analysis revealed no significant correlation between platinum desensitization therapy and progression-free survival (PFS) or overall survival (OS) of patients (*P* < 0.05). However, the subgroup analysis demonstrated that the success or failure of platinum desensitization therapy significantly impacted the OS of patients who were platinum-sensitive recurrence. The patients who had successful desensitization therapy had a superior OS.

**Conclusion:**

Five-step platinum desensitization therapy has potential application value in patients who were platinum-sensitive recurrence after first-line treatment but may bear the risk of severe allergic reactions.

## Highlights

- Platinum desensitization therapy has potential value for platinum-sensitive recurrence ovarian cancer.
- Platinum desensitization therapy may bear the risk of severe allergic reactions.
- Five-step platinum desensitization therapy is safe.

## 1 Introduction

Platinum-based chemotherapy is the first-line chemotherapy regimen for the initial treatment of epithelial ovarian cancer (1). The incidence of allergic reactions caused by these agents has increased and attracted attention with the widespread clinical application of platinum-based chemotherapy agents. Studies showed that the risk of allergic reactions increased significantly with the increase in drug treatment courses, and most of the reactions occurred in patients who had received more than five or six courses of treatment (2). Markman et al. (3) showed that the incidence of carboplatin allergy in the first five courses was less than 1% in patients who received carboplatin chemotherapy for the first time, but increased to 6.5% in the sixth course. It was as high as 27% in patients receiving more than seven courses. The risk of allergic reactions increased significantly in recurrent patients or patients who received eight or more courses of treatment, with an incidence rate of up to 44% (4–7).

Platinum desensitization therapy has become an essential clinical technique for reintroducing platinum-based chemotherapy and is widely used in clinical practice (8–11). Rapid drug desensitization has become the standard treatment for patients with platinum drug allergies since the early, 2000s (12–14). Eroglu et al. (14) reported a 6-h, 12-step desensitization protocol for carboplatin allergy, including pretreatment with leukotriene receptor antagonists, antihistamines, and corticosteroids, as well as extended infusion time. In their study, 186 eligible patients were included, with 155 (83%) receiving platinum-based treatment and 104 (56%) completing three or more cycles of therapy during the desensitization period. Overall, the patients completed 694 cycles. Further, 79 (42%) patients experienced breakthrough hypersensitivity reactions during desensitization, with 4 patients requiring epinephrine and 84 (45%) discontinued desensitizing agents due to disease progression. Paksoy et al. (15) demonstrated that the 6-h, 12-step rapid drug desensitization protocol was safe and effective, with a total survival time of 42.2 months (range: 25.3–59.1 months) after the first desensitization treatment (0S2). The 1-, 2-, and 5-year survival rates were 92.6%, 75.6%, and 47.2%, respectively. The objective response rate was 78.5%. However, various complex clinical confounding factors may affect the long-term prognosis of patients, making it unclear whether desensitization treatment can significantly improve the progression-free survival (PFS) or overall survival (OS) of patients with ovarian cancer. Multivariate analysis can further eliminate the influence of other confounding factors, thereby determining the correlation between predictor variables and response variables. In addition, it is easier to find problems by comparing the results of Univariate and Multivariate. If the results of Multivariate analysis and Univariate analysis are consistent, the conclusion is more stable and easier to explain. Therefore, this study aimed to use multifactorial analysis to investigate the impact of platinum desensitization outcomes on the prognosis of patients with ovarian cancer, providing a basis for better-serving patients with platinum desensitization therapy.

## 2 Materials and methods

### 2.1 Research participants

This study was focused on patients with high-grade serous adenocarcinoma of the ovary (HGSAO) who developed a platinum allergy during cancer treatment and received concurrent desensitization therapy from January, 2016 to December, 2020. The inclusion criteria were as follows: patients aged 18 years or older, diagnosed with HGSAO by two independent pathologists based on the pathological slides, having platinum allergy during initial treatment or at recurrence, and receiving concurrent platinum desensitization therapy. Platinum agents included cisplatin, carboplatin, oxaliplatin, and nedaplatin. The exclusion criteria were as follows: patients who developed a platinum allergy during treatment but did not receive platinum desensitization therapy or those without HGSAO.

The scholars collected the clinical information of the patients through the hospital’s electronic health record system, including age, pathological type, the International Federation of Gynecology and Obstetrics (FIGO) stage, neoadjuvant chemotherapy, number of chemotherapy cycles, surgical outcomes (R0, R1, or R2), maintenance therapy, allergy medication (cisplatin, carboplatin, oxaliplatin, or nedaplatin), desensitization medication (cisplatin, carboplatin, oxaliplatin, or nedaplatin), desensitization outcomes (success or failure), severity of allergy (mild, moderate, or severe), and prognostic outcomes (OS and PFS1: first recurrence; PFS2: first recurrence after desensitization).

### 2.2 Treatment

All patients received initial surgical treatment (comprehensive surgical staging) or interval cytoreductive surgery (after neoadjuvant chemotherapy), followed by platinum-based chemotherapy. All patients voluntarily underwent genetic testing and maintenance therapy and were followed up regularly in outpatient clinics. Platinum-sensitive recurrent patients received platinum-based chemotherapy. Interval cytoreductive surgery was performed before chemotherapy in patients with a chance of achieving R0 after surgery. Personalized treatment was adopted in platinum-resistant patients based on the National Comprehensive Cancer Network (NCCN) guidelines.

**Figure 1:**
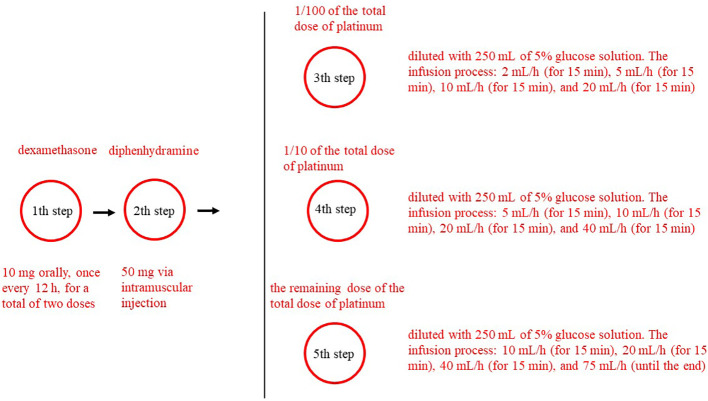
The platinum desensitization protocol.

The platinum desensitization protocol is shown in **Figure 1**. The platinum desensitization protocol used in this study consisted of five-step. The first step involved administering dexamethasone 10 mg orally, once every 12 h, for a total of two doses. The second step involved administering diphenhydramine 50 mg via intramuscular injection. The third step to the fifth step involved preparing three different concentrations of platinum solution. The third step involved 1/100 of the total dose of platinum, and diluted with 250 mL of 5% glucose solution. The infusion process for the first concentration of platinum was as follows: 2 mL/h (for 15 min), 5 mL/h (for 15 min), 10 mL/h (for 15 min), and 20 mL/h (for 15 min). The fourth step involved 1/10 of the total dose of platinum, and diluted with 250 mL of 5% glucose solution. For the second concentration of platinum, it was 5 mL/h (for 15 min), 10 mL/h (for 15 min), 20 mL/h (for 15 min), and 40 mL/h (for 15 min). The fifth step involved the remaining dose of the total dose of platinum, and diluted with 250 mL of 5% glucose solution. For the third concentration of platinum, it was 10 mL/h (for 15 min), 20 mL/h (for 15 min), 40 mL/h (for 15 min), and 75 mL/h (until the end). Desensitization treatment was immediately stopped after the occurrence of allergic reaction symptoms.

The evaluation of patients who experience allergic reactions includes categorization of reactions as mild (cutaneous symptoms), moderate (cutaneous, respiratory, and gastrointestinal involvement), and severe (changes in vital signs, syncope, seizures, and cardiac or respiratory arrest). Successful desensitization is defined as symptom-free during the desensitization. If any degree of allergic reaction occurs during the desensitization process, it is considered a desensitization failure.

We have emergency response plans for severe allergic reactions. If a severe allergic reaction occurs when a patient undergoes chemotherapy or desensitization treatment, we will immediately initiate an emergency process. This process consists of multiple members, including oncologists, internists, anesthesiologists, emergency doctors, nurses, workers, etc. When an allergic reaction occurs, treatment must be prompt and timely, every second counts. Stop the medication immediately, lie down, inhale oxygen, and keep warm. Closely observe and record blood pressure, pulse, respiration, urine output, etc. Immediately subcutaneously inject 0.5 to 1 ml of 0.1% epinephrine hydrochloride. If the symptoms are not relieved, 0.1% epinephrine hydrochloride 0.5 ml or 0.1% epinephrine hydrochloride 0.5ml + 50% GS 40 ml intravenously can be injected subcutaneously every 10 to 30 minutes until out of the dangerous period. At the same time, dexamethasone 20mg + 50% GS 40ml intravenously is given, followed by dexamethasone 20mg + 5% GS 500ml intravenous infusion, or hydrocortisone 200 ~ 300mg + 5% ~ 10% glucose intravenous infusion. Antihistamine Drugs such as promethazine hydrochloride 25 to 50 mg or 10% calcium gluconate 10 to 20 ml are diluted and then injected intravenously or diphenhydramine 40 mg is injected intramuscularly. After the above treatment, if the condition does not improve and the blood pressure does not rise, it is necessary to establish 1 to 2 infusion channels in time to supplement the blood volume and give rescue drugs intravenously in a timely manner. If the blood pressure still does not rise, consider vasopressors, such as dopamine, alamin, norepinephrine, etc. When shock is accompanied by tracheospasm, immediately intravenously inject aminophylline 0.25, dexamethasone 10 mg, and 50% GS 20 ml, followed by intravenous infusion of 10% GS 500 ml, aminophylline 0.5mg, and dexamethasone 10 mg. In case of cardiac arrest, 1 ml of 0.1% epinephrine is injected intracardially, and external or intrathoracic cardiac massage is performed.

### 2.3 Statistical analysis

Statistical Analysis System (SAS) 9.2 was used for the statistical analysis of the data. The continuous data were presented as mean ± standard deviation, while the count data were presented as percentages. A *P* value of <0.05 indicated a statistically significant difference. The hazard ratio (HR) was used to evaluate the impact of variables on the outcome event (death or recurrence), where an HR point estimate greater than 1 indicated an increased risk of the outcome event with the increase in the variable value, and vice versa. The single-factor statistical modeling was used for preliminary evaluation and screening of variables, and multiple-factor statistical modeling was performed by incorporating variables with clinical significance.

Single-factor statistical modeling is used for preliminary evaluation and screening of variables, and multi-factor statistical modeling is implemented by incorporating clinically significant variables based on clinical considerations. P<0.05 indicates that the difference is statistically significant, but it is not an absolute standard. Failure to meet statistical significance does not mean that it is without clinical significance. After all, the overall sample size is limited, and statistical power does not necessarily support the conclusion of statistical significance. **Tables 1**, **2** are examples. The first recurrence will be modeled by considering factors in turn. FIGO stage, neoadjuvant chemotherapy, number of chemotherapy, and preliminary assessment of allergy drug categories may have a certain impact on recurrence. Comprehensive clinical considerations, these factors were incorporated into a multi-factor model for unified modeling, and it was found that neoadjuvant chemotherapy, the more severe the allergy, the number of chemotherapy, and allergy drugs have statistically significant effects on the duration of recurrence. At the same time, surgery also has a significant impact on the duration of recurrence. There is likely to be an impact. Other factors showed no statistically significant effects, possibly due to insufficient statistical power due to insufficient samples.

**Table 1:** Cross-tabulation of recurrence before and after desensitization treatment.

		Relapse distribution after desensitization therapy (%)				
		unrecurrence	>6 months	<6 months	progression	total
Recurrence at the end of follow-up	unrecurrence	9 (90.0%)	0 (0.0%)	0 (0.0%)	1 (10.0%)	10 (10.9%)
	>6 months	24 (35.3%)	22 (32.3%)	1 (1.5%)	21 (30.9%)	68 (73.9%)
	<6 months	3 (30.0%)	1 (10.0%)	3 (30.0%)	3 (30.0%)	10 (10.9%)
	progression	0 (0.0%)	0 (0.0%)	0 (0.0%)	4 (100%)	4 (4.4%)
	total	36 (39.1%)	23 (25.0%)	4 (4.4%)	29 (31.5%)	92 (100%)

**Table 2:** Multivariate analysis of influence on Patient’s prognosis.

variable types		DF	PE	STD ERR	X^2^	P	HR	HR 95%CI	
OS	FIGO	1	0.32577	0.35054	0.8636	0.3527	1.385	0.697	2.753
	neoadjuvant chemotherapy	1	0.78097	0.36020	4.7010	0.0301	2.184	1.078	4.424
	allergic	1	0.25763	0.34749	0.5497	0.4584	1.294	0.655	2.557
	Surgical outcome	1	0.11328	0.22536	0.2527	0.6152	1.120	0.720	1.742
	Maintenance treatment	1	-0.66046	0.45183	2.1367	0.1438	0.517	0.213	1.252
PFS1	FIGO	1	-0.12379	0.23182	0.2852	0.5933	0.884	0.561	1.392
	neoadjuvant chemotherapy	1	0.92902	0.28269	10.8000	0.0010	2.532	1.455	4.407
	allergic	1	0.54727	0.21326	6.5856	0.0103	1.729	1.138	2.625
	Chemotherapy cycles	1	0.80586	0.20833	14.9632	0.0001	2.239	1.488	3.368
	Surgical outcome	1	0.31210	0.17067	3.3443	0.0674	1.366	0.978	1.909
	Maintenance treatment	1	-0.34536	0.22409	2.3751	0.1233	0.708	0.456	1.098
	Allergy drug	1	0.35025	0.15831	4.8950	0.0269	1.419	1.041	1.936
PFS2	FIGO	1	0.65887	0.39556	2.7745	0.0958	1.933	0.89	4.196
	neoadjuvant chemotherapy	1	-0.40879	0.52347	0.6098	0.4349	0.664	0.238	1.854
	allergic	1	-0.14548	0.41474	0.1230	0.7258	0.865	0.384	1.949
	Chemotherapy cycles	1	-0.43906	0.34058	1.6619	0.1974	0.645	0.331	1.257
	desensitization outcome	1	0.95401	1.25883	0.5743	0.4485	2.596	0.220	30.608
	Surgical outcome	1	0.26510	0.28226	0.8821	0.3476	1.304	0.750	2.267
	Maintenance treatment	1	1.06397	0.36182	8.6469	0.0033	2.898	1.426	5.889
	Desensitization drugs	1	0.11367	0.29666	0.1468	0.7016	1.120	0.626	2.004

FIGO, The International Federation of Gynecology and Obstetrics; PFS, progression-free survival; OS, overall survival; DF, degree of freedom; PE, Parameter Estimation; STD ERR, standard Error; HR, Hazard Ratio; CI, confidence interval.

## 3 Results

### 3.1 Basic characteristics of the study participants

**Figure 2:**
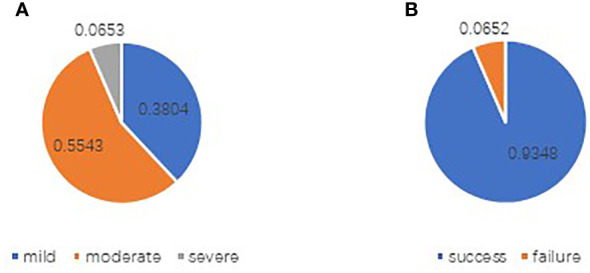
Distribution of allergy levels in patients with platinum allergy **(A)** and outcomes of desensitization treatment **(B)**.

**Figure 3:**
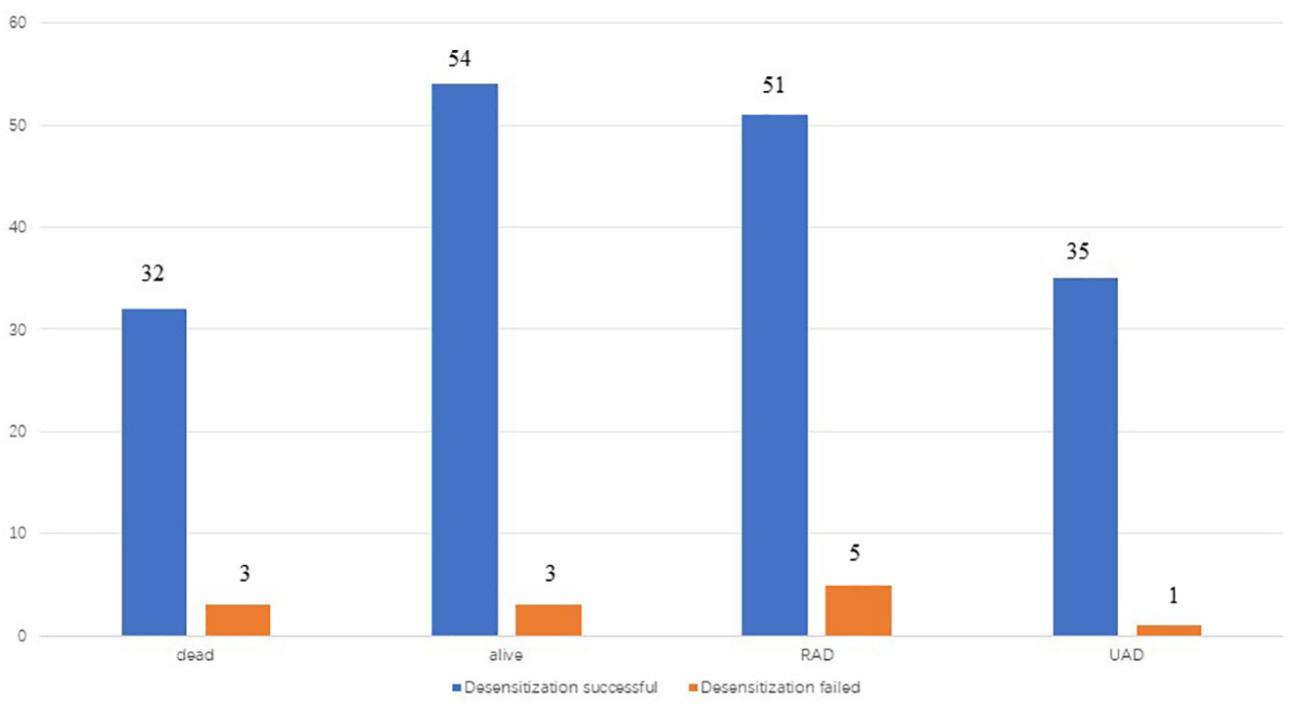
recurrence and death of patients in two groups(desensitization successful and desensitization failed) by the end of follow-up.

The basic characteristics of the study participants are shown in **Tables 3**, **4**, and **Figures 2**, **3**. A total of, 1592 patients with HGSAO were treated over 5 years, with 127 (7.98%) experiencing platinum-based agent allergies. Among the 92 patients with HGSAO who met the inclusion criteria, 35 (38.0%) experienced mild allergic reactions, 51 (55.4%) experienced moderate reactions, and 6 (6.5%) experienced severe reactions. A total of 552 desensitization treatments were administered to the 92 patients, with 6 cases of failures. The success rates for desensitization treatment were 93.5% (86/92) and 98.9% (546/552). Of the 92 allergic patients, 19 (20.7%) were allergic to cisplatin, 55 (59.8%) to carboplatin, 12 (13.0%) to oxaliplatin, and 6 (6.5%) to nedaplatin. The desensitization agents used in the 92 desensitization-treated patients were cisplatin in 1 case (1.1%), carboplatin in 65 cases (70.7%), oxaliplatin in 15 cases (16.3%), and nedaplatin in 11 cases (11.9%).

**Table 3:** The basic characteristics of patients.

variable types	n (%)
FIGO stage	
FIGO I stage	5 (5.4%)
FIGO II stage	10 (10.9%)
FIGO III stage	66 (71.7%)
FIGO IV stage	11 (12.0%)
neoadjuvant chemotherapy	
Y	55 (59.8%)
N	37 (40.2%)
Maintenance treatment	
no	76 (82.6%)
before desensitization	11 (12.0%)
after desensitization	5 (5.4%)
chemotherapy cycles	
Low-5	16 (17.4%)
5-10	26 (28.3%)
10-High	49 (53.3%)
Surgical outcome	
R0	60 (65.2%)
R1	17 (18.5%)
R2	15 (16.3%)

R0: No cancer remain; R1: All large lesions have been removed, and there are cancer on the resection margin under the microscope; R2: Tumor remains visible to the eyes; FIGO: The International Federation of Gynecology and Obstetrics.

**Table 4:** Basic characteristics related to desensitization.

Variable types	n (%)
Allergy medicine	
Cisplatin	19 (20.7%)
Carboplatin	55 (59.8%)
Oxaliplatin	12 (13.0%)
Nedaplatin	6 (6.5%)
Desensitization drugs	
Cisplatin	1 (1.1%)
Carboplatin	65 (70.7%)
Oxaliplatin	15 (16.3%)
Nedaplatin	11 (11.9%)
Desensitization outcome	
Desensitization successful	86 (93.5%)
Desensitization failed	6 (6.5%)

Six cases of desensitization failure, one experienced mild allergic reactions, five experienced severe allergic reactions during desensitization and required rescue treatment, such as adrenaline, to alleviate symptoms. Desensitization failure in 6 patients occurred after multiple desensitizations.

The patients were followed up for an average of 48.9 months during the study. Further, 78 patients (84.8%) had experienced recurrence by the end of the follow-up period, while 4 patients (4.4%) had experienced disease progression during initial treatment. Among the 78 patients who experienced recurrence, 68 were platinum-sensitive and 10 were platinum-resistant. Moreover, 34 patients (37.0%) had deceased by the end of the follow-up period and all 34 patients died of ovarian cancer. A total of 36 patients (39.1%) did not experience recurrence, 23 patients (25.0%) experienced recurrence after stopping the therapy for more than 6 months, 4 patients (4.4%) experienced recurrence within 6 months of stopping the therapy, and 29 patients (31.5%) experienced disease progression during the period from desensitization treatment to the end of follow-up.

### 3.2 Effects of desensitization therapy on PFS or OS

The risk factors for PFS or OS included the FIGO stage, neoadjuvant chemotherapy, severity of the allergy, number of chemotherapy cycles, desensitization outcome, surgical outcome, maintenance therapy, and allergy agent. The univariate analysis showed that the desensitization outcome did not affect the initial PFS outcome (PFS 1), the PFS outcome (PFS 2) after desensitization treatment and OS in patients with ovarian cancer (*P* > 0.05). The results of the multivariate analysis were consistent with those of the univariate analysis. The details are presented in **Table 2**.

### 3.3 Effects of desensitization therapy on the prognosis of patients with different types of recurrence

The risk factors for the prognosis of patients with different types of recurrence included the FIGO stage, neoadjuvant chemotherapy, severity of the allergy, number of chemotherapy cycles, desensitization outcome, surgical outcome, maintenance treatment, and allergy agent. The univariate and multivariate analyses revealed that the desensitization outcomes did not affect the OS outcomes in platinum-resistant recurrent patients after initial treatment or patients with progressive ovarian cancer (*P* > 0.05). However, it significantly affected the OS outcomes of platinum-sensitive recurrent patients, with successful desensitization leading to better OS outcomes (*P* < 0.05). The details are presented in **Table 5**.

**Table 5:** Subgroup analysis: Multivariate analysis of influence on Patient’s prognosis (platinum sensitive relapse).

variable types		DF	PE	STD ERR	X^2^	P	HR	HR 95%CI	
PFS2	FIGO	1	0.14757	0.49896	0.0875	0.7674	1.159	0.436	3.082
	neoadjuvant chemotherapy	1	0.01693	0.62015	0.0007	0.9782	1.017	0.302	3.429
	allergic	1	-0.65006	0.50724	1.6424	0.2000	0.522	0.193	1.411
	chemotherapy cycles	1	-0.86049	0.41662	4.2659	0.0389	0.423	0.187	0.957
	desensitization outcome	1	0.52742	1.25318	0.1771	0.6739	1.695	0.145	19.759
	Surgical outcome	1	0.24655	0.29059	0.7199	0.3962	1.280	0.724	2.262
	Maintenance treatment	1	1.08510	0.36610	8.7849	0.0030	2.960	1.444	6.066
	Desensitization drugs	1	0.36415	0.37561	0.9399	0.3323	1.439	0.689	3.005
OS	FIGO	1	-0.0299	0.51491	0.0034	0.9537	0.971	0.354	2.663
	neoadjuvant chemotherapy	1	1.72302	0.54462	10.009	0.0016	5.601	1.926	16.288
	allergic	1	0.27486	0.42365	0.4209	0.5165	1.316	0.574	3.020
	chemotherapy cycles	1	0.23943	0.35939	0.4438	0.5053	1.271	0.628	2.570
	desensitization outcome	1	-1.7540	0.72829	5.8003	0.0160	0.173	0.042	0.721
	Surgical outcome	1	-0.0777	0.30764	0.0638	0.8005	0.925	0.506	1.691
	Maintenance treatment	1	-0.7743	0.51750	2.2389	0.1346	0.461	0.167	1.271
	Desensitization drugs	1	-0.3111	0.41561	0.5604	0.4541	0.733	0.324	1.654

FIGO, The International Federation of Gynecology and Obstetrics; PFS, progression-free survival; OS, overall survival; DF, degree of freedom; PE, Parameter Estimation; STD ERR, standard Error; HR, Hazard Ratio; CI, confidence interval.

## 4 Discussion

### 4.1 Principal finding

Platinum desensitization therapy has potential application value in patients who were platinum-sensitive recurrence after first-line treatment but may bear the risk of severe allergic reactions.

### 4.2 Results in the context of what is known

Platinum agents can cause type I reactions, cytokine release reactions, and mixed reactions. Evidence supporting an immunoglobulin E (IgE)-mediated hypersensitivity reaction (HSR) mechanism of platinum agents was first reported in refinery workers exposed to platinum salts, followed by the detection of carboplatin-specific IgE (12). Most reactions occurred immediately, during, or within hours after infusion. The clinical features of platinum-based agent allergies were diverse and varied in severity, with most presenting as mild-to-moderate reactions such as rash, itching, chest tightness, and palpitations, and a few presenting as severe reactions such as difficulty breathing or even anaphylactic shock. No studies to date reported deaths due to platinum agent allergies.

Rapid drug desensitization (RDD) is a treatment modality that renders mast cells less responsive, thereby protecting patients from allergic reactions. In IgE-sensitized patients, desensitization inhibits the mechanisms of mast cell activation (12). The NCCN guidelines recommend desensitization as an option for patients who have experienced drug reactions (1). Platinum desensitization therapy has also been successful in many cases. Confino-Cohen et al. (16) reported a safe and effective desensitization therapy for patients with tumors who were allergic to carboplatin. Among 228 patients with ovarian cancer or primary peritoneal cancer and 26 patients with endometrial serous papillary carcinoma who received carboplatin monotherapy or carboplatin-based combination chemotherapy, patients who developed immediate hypersensitivity reactions to carboplatin were administered a carboplatin skin test. Further, the patients who had a positive skin test were given carboplatin desensitization therapy for 6 h in each subsequent treatment. The desensitization therapy involved preparing four concentrations of carboplatin solution, with the first three containing 1/1000, 1/100, and 1/10 total dose of carboplatin, respectively, diluted in 150 mL of 5% glucose, and the fourth containing the remaining carboplatin. The infusion started with the 1/1000 carboplatin solution, and the infusion time for each concentration was more than 90 min. If the previous concentration was infused successfully, the next higher concentration was infused immediately. Twenty-three patients were allergic to carboplatin and had positive skin tests. Twenty patients received desensitization therapy while continuing carboplatin chemotherapy. Only one patient experienced mild skin rash in the first desensitization therapy and discontinued the treatment, whereas the other 19 patients had no adverse reactions and tolerated 80 courses of desensitization chemotherapy. Lee et al. (17) reported a 6-h, 12-step desensitization protocol for carboplatin allergy. The desensitization protocol involved preparing three concentrations of carboplatin solution, with the first two containing 1/100 and 1/10 total dose of carboplatin, respectively, diluted in 250 mL of 5% glucose solution, and the third containing the remaining carboplatin. The first concentration of carboplatin was used in steps 1–4, the second concentration in steps 5–8, and the third in steps 9–12. In the first 11 steps, the carboplatin infusion speed was adjusted every 15 min (doubled) in each step. The infusion started with 1/100 carboplatin, and the infusion time for each concentration was more than 90 min. Ten patients who received carboplatin monotherapy or carboplatin-based combination chemotherapy were treated, including eight patients with ovarian cancer, 1 with primary peritoneal cancer, and 1 with endometrial cancer. All 10 patients successfully completed 35 desensitization courses against carboplatin, with 31 courses showing no reactions. Four patients developed symptoms during the first (*n* = 3) or third (*n* = 1) desensitization courses, but repeat injections were tolerated without further reactions. The desensitization protocols for two patients who had developed cutaneous reactions were adjusted, while no adjustments were made for one patient who had developed a mild rash. All three patients could tolerate subsequent desensitization therapy without adverse reactions. The fourth patient had a reaction during desensitization therapy and could not receive further carboplatin treatment due to disease progression. Of the five patients who underwent carboplatin skin testing, four had positive blisters and erythema. One patient had a negative skin test for carboplatin after desensitization therapy. Kokabu et al. (18) reported successful nedaplatin desensitization, suggesting that the nedaplatin desensitization regimen could be a new alternative for hypersensitivity reactions to platinum-based agents. In this study, a 5-step platinum desensitization protocol was used for 92 patients, and 552 desensitization treatments were performed for platinum agents including cisplatin, carboplatin, oxaliplatin, and nedaplatin. Only six treatments failed, resulting in a high success rate of 98.9%. The results indicated that platinum desensitization therapy was effective, and all platinum agents could be desensitized using the five-step desensitization protocol. However, among the six cases of desensitization failure, five experienced severe allergic reactions during desensitization and required rescue treatment, such as adrenaline, to alleviate symptoms. We have emergency response plans for anaphylactic shock. After rescue and treatment, all patients who failed desensitization were significantly relieved. Although the success rate of desensitization was high, the potential risk of severe allergic reactions in patients who failed desensitization might be high too.

Platinum-based chemotherapy is effective in ovarian cancer treatment, with an initial response rate as high as 60%–80% (19). Whether platinum desensitization treatment is associated with the prognosis of patients with ovarian cancer and whether it is worth bearing the potential risk of fatal allergic reactions during desensitization treatment after an allergic reaction to platinum chemotherapy agents remain unclear due to the lack of clinical data. This study retrospectively analyzed the impact of platinum desensitization on the prognosis of patients with ovarian cancer, taking into account confounding factors such as patient’s FIGO stage, neoadjuvant chemotherapy, surgical outcome, maintenance therapy, and severity of allergic reactions. The present study revealed that desensitization treatment improved the prognosis of platinum-sensitive recurrent patients, but did not improve the prognosis of platinum-resistant recurrent patients or patients with disease progression during initial treatment. The desensitization treatment may provide survival benefits to platinum-resistant recurrent patients who experience a platinum allergy reaction, but the potential risk of severe allergic reactions caused by desensitization treatment needs to be considered.

### 4.3 Clinical implications and research implications

Various complex clinical confounding factors may affect the long-term prognosis of patients, making it unclear whether desensitization treatment can significantly improve the progression-free survival (PFS) or overall survival (OS) of patients with ovarian cancer. Therefore, this study aimed to use multifactorial analysis to investigate the impact of platinum desensitization outcomes on the prognosis of patients with ovarian cancer, providing a basis for better-serving patients with platinum desensitization therapy. The success rate of desensitization in 6 patients with severe allergic reactions was 100%. Was there a relationship between the degree of allergy reaction and the successful rate of desensitization? Because the sample size was too small, we were unable to answer this question. A large sample study is needed. We are planning a multicenter, large-sample, real-world study.

### 4.4 Strengths and limitations

This study was a single-center retrospective clinical study with small sample size and a long treatment time span. The influence of patient’s genetic status and other unpredictable confounding factors was not investigated. Therefore, the results of this study should be evaluated with caution. Notably, this study was novel in exploring the application value of platinum desensitization in ovarian cancer through multivariate analysis and analyzing various outcomes of platinum allergy and desensitization at the same time, demonstrating significant clinical guidance value.

## 5 Conclusions

In summary, the five-step platinum desensitization protocol was found to be safe and effective. The platinum-sensitive recurrent patients may benefit from platinum desensitization treatment, but the potential risk of serious allergic reactions should be considered. Further, large-sample prospective high-quality clinical studies are needed to confirm these findings.

## Data availability statement

The original contributions presented in the study are included in the article/supplementary material. Further inquiries can be directed to the corresponding author.

## Ethics statement

The studies involving humans were approved by Medical Ethics Committee of West China Second University Hospital, Sichuan University. The studies were conducted in accordance with the local legislation and institutional requirements. The ethics committee/institutional review board waived the requirement of written informed consent for participation from the participants or the participants’ legal guardians/next of kin because exempt from signing informed consent by Medical Ethics Committee.

## Author contributions

KL: Conceptualization, Formal Analysis, Methodology, Software, Writing – original draft, Writing – review & editing. RY: Conceptualization, Formal Analysis, Funding acquisition, Investigation, Methodology, Project administration, Resources, Writing – review & editing.
